# The Stimulatory Effects of Intracellular α-Synuclein on Synaptic Transmission Are Attenuated by 2-Octahydroisoquinolin-2(1H)-ylethanamine

**DOI:** 10.3390/ijms222413253

**Published:** 2021-12-09

**Authors:** Alejandra E. Ramirez, Eduardo J. Fernández-Pérez, Nicol Olivos, Carlos F. Burgos, Subramanian Boopathi, Lorena Armijo-Weingart, Carla R. Pacheco, Wendy González, Luis G. Aguayo

**Affiliations:** 1 Laboratory of Neurophysiology, Department of Physiology, Faculty of Biological Sciences, Universidad de Concepción, 160-C, Concepción 4030000, Chile; alejandra.ramirez@florey.edu.au (A.E.R.); niolivos@udec.cl (N.O.); caburgos@udec.cl (C.F.B.); lorena.armijo@gmail.com (L.A.-W.); carlapacheco@udec.cl (C.R.P.); 2 Instituto de Ciencias Físicas, Universidad Nacional Autónoma de México, Cuernavaca 62210, Mexico; boopathi@icf.unam.mx; 3 Center for Bioinformatics, Simulations and Modeling, The Center for Bioinformatics and Molecular Simulations (CBSM), University of Talca, Talca 3530000, Chile; wgonzalez@utalca.cl; 4 Millennium Nucleus of Ion Channels-Associated Diseases, The Center for Bioinformatics and Molecular Simulations (CBSM), University of Talca, Talca 3530000, Chile; 5 Programa de Neurociencia, Psiquiatría y Salud Mental, Anatomy Building, Faculty of Medicine, Universidad de Concepción, Concepción 4030000, Chile

**Keywords:** Parkinson’s disease, α-Synuclein, oligomers, synapsis, miniature synaptic currents, synucleinopathies

## Abstract

α-Synuclein (αSyn) species can be detected in synaptic boutons, where they play a crucial role in the pathogenesis of Parkinson’s Disease (PD). However, the effects of intracellular αSyn species on synaptic transmission have not been thoroughly studied. Here, using patch-clamp recordings in hippocampal neurons, we report that αSyn oligomers (αSynO), intracellularly delivered through the patch electrode, produced a fast and potent effect on synaptic transmission, causing a substantial increase in the frequency, amplitude and transferred charge of spontaneous synaptic currents. We also found an increase in the frequency of miniature synaptic currents, suggesting an effect located at the presynaptic site of the synapsis. Furthermore, our in silico approximation using docking analysis and molecular dynamics simulations showed an interaction between a previously described small anti-amyloid beta (Aβ) molecule, termed M30 (2-octahydroisoquinolin-2(1H)-ylethanamine), with a central hydrophobic region of αSyn. In line with this finding, our empirical data aimed to obtain oligomerization states with thioflavin T (ThT) and Western blot (WB) indicated that M30 interfered with αSyn aggregation and decreased the formation of higher-molecular-weight species. Furthermore, the effect of αSynO on synaptic physiology was also antagonized by M30, resulting in a decrease in the frequency, amplitude, and charge transferred of synaptic currents. Overall, the present results show an excitatory effect of intracellular αSyn low molecular-weight species, not previously described, that are able to affect synaptic transmission, and the potential of a small neuroactive molecule to interfere with the aggregation process and the synaptic effect of αSyn, suggesting that M30 could be a potential therapeutic strategy for synucleinopathies.

## 1. Introduction

Synucleinopathies are defined as a group of diseases characterized by the accumulation of α-Synuclein (αSyn) in neurons, nerve fibers, and glial cells. Diseases such as Parkinson’s Disease (PD) and dementia with Lewy bodies (DLB) share pathological features that are characterized by the presence of neuronal lesions called Lewy bodies [1]. A large body of evidence indicates that αSyn plays a pivotal role in the pathogenesis of synucleinopathies [2,3,4]. The aggregation of αSyn monomers into amyloid fibrils has been suggested as the disease-causative toxic mechanism, with αSyn oligomeric intermediates being the main culprits of this neurotoxicity [4,5]. In fact, mutations [6,7,8] and αSyn gene multiplications [9] are linked to an increased susceptibility of αSyn aggregation [8,9,10] and are associated with the early onset of PD [7,8]. However, the role of αSyn and its specific neurodegenerative effects are not yet fully understood.

The inhibition of the ubiquitin-proteasome system [11], mitochondrial dysfunction [12], the production of reactive oxygen [13], and changes in synaptic vesicle release [14] are among the processes explaining the toxic role of intracellular αSyn oligomers. Moreover, recent studies have described the detrimental effect of intracellularly delivered αSyn oligomers administered through a patch pipette on neuronal physiology [15,16,17]. This experimental strategy allows the rapid examination of potential underlying toxic effects of abnormal intracellular αSyn accumulation on synaptic function, which could be relevant in the early stages of the disease. Additionally, understanding the involvement of intracellular αSyn oligomers in early cellular events that lead to neurodegeneration is critical for the development of novel therapeutic strategies for synucleinopathies.

In this context, small molecules have demonstrated therapeutic efficacy in interfering with αSyn aggregation in different PD animal models [18]. The high-throughput screening of large chemical libraries has allowed the identification of novel molecules that act as inhibitors of αSyn aggregation [19,20]. In addition, small molecules have the advantage of crossing the blood–brain barrier (BBB) and avoiding collateral immunological reactions [21].

Recently, we identified a new compound, referred to as M30 (2-octahydroisoquinolin-2(1H)-ylethanamine), that inhibits Aβ-induced neurotoxicity and prevents monomer aggregation through its interaction with hydrophobic domains that are present in the carboxyl terminus of Aβ [22]. This domain shares striking sequence similarity with the central domain (61–95) of αSyn, which is highly hydrophobic, and it is involved in αSyn aggregation when acquiring a β-sheet structure [23]. Thus, we hypothesized that M30 might also interact with αSyn and subsequently hinder its aggregation and cellular actions.

In the present study, we sought to characterize the direct effect of low-molecular-weight αSyn oligomers (αSynO) on synaptic activity and a potential protective effect of M30. Therefore, we biochemically and structurally characterized αSyn species that formed after a standardized oligomerization process that was previously published by our group [24]. The resulting species were associated with neuronal membranes, increased membrane conductance and intracellular calcium, leading to augmentation in synaptic transmission. Here, we found that the acute intracellular exposure of αSynO also enhanced neurotransmission. The data show that M30 reduced the effect of αSynO, suggesting that it may serve as a potential therapeutic agent against synucleinopathies.

## 2. Results

### 2.1. Characterization of αSyn Oligomers

**Figure 1:**
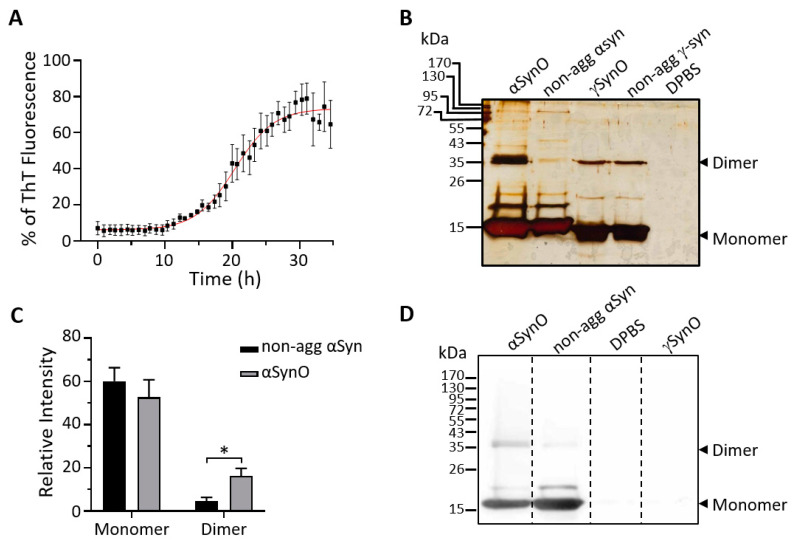
Aggregation kinetics and the biochemical characterization of αSyn species. (**A**) Aggregation kinetics of 32 µM α-Synuclein (αSyn) in presence of 24 µM thioflavin T (ThT) in DPBS of pH 4.0, at 37 °C for 35 h. Fibril formation was monitored by an increase in ThT fluorescence at 485 nm. The graph summarizes the quantification of ThT fluorescence, adjusted to a Boltzmann sigmoidal curve. Values are expressed as a percentage of the maximum ThT fluorescence. The graph represents the mean ± SEM (*n* = 3 independent experiments). (**B**) The vehicle (DPBS, pH 4.0), non-aggregated (non-agg) αSyn and γSyn, and αSyn and γSyn after 24 h of the oligomerization process (αSynO and γSynO, respectively) were analyzed on 15% SDS polyacrylamide denaturing silver-stained gel. (**C**) The graph summarizes relative changes in the levels of αSyn species normalized within the same lane for the conditions described in (**B**). Bars represent the mean ± SEM (*n* = 5 independent experiments, * *p* < 0.05; unpaired *t*-test). (**D**) The vehicle (DPBS, pH 4.0), non-agg αSyn, αSynO, and γSynO samples were analyzed by Western blot with an anti-αSyn antibody (syn-211).

We first monitored the oligomerization kinetics of αSyn using thioflavin T (ThT), a fluorescent reporter of amyloid fibril formation [25]. Hence, 32 μM of recombinant human αSyn (1–140) was incubated at pH 4.0 and 37 °C in the presence of 24 μM of ThT for 35 h (Figure 1A). The aggregation of αSyn showed a typical sigmoidal-shaped ThT fluorescence curve. Notably, αSyn exhibited slower aggregation kinetics when compared to Aβ_40_, which presented an overall increase in ThT fluorescence after 5 h (Supplementary Figure S1).

We previously reported that the in vitro αSyn assembles into soluble low-molecular-weight oligomers [24]. To determine whether the accumulation of β-sheet conformation, as reported by ThT fluorescence, was consistent with the formation of αSyn oligomers (αSynO), 60 μM of αSyn or γSyn were vertically stirred at 800 rpm at 37 °C for 24 h. After oligomerization, samples were centrifuged, and the supernatants were run on SDS denaturing gels and visualized with silver staining. The αSynO banding pattern (Figure 1B) revealed a significant increase in low-molecular-weight oligomers, specifically dimers (dimers ~35 kDa), compared to non-aggregated (non-agg) αSyn (Figure 1C). In addition, Western blot analysis was performed using an anti-αSyn antibody directed against the amino acids 121–125 of human αSyn (syn-211). In agreement with the result observed using silver staining (Figure 1B), there was a clear increase in the signal for the dimeric species in the αSynO sample (Figure 1D). Moreover, the antibody immunoreactivity was positive for αSyn but not for γSyn, indicating its specificity for the αSyn protein.

**Figure 2:**
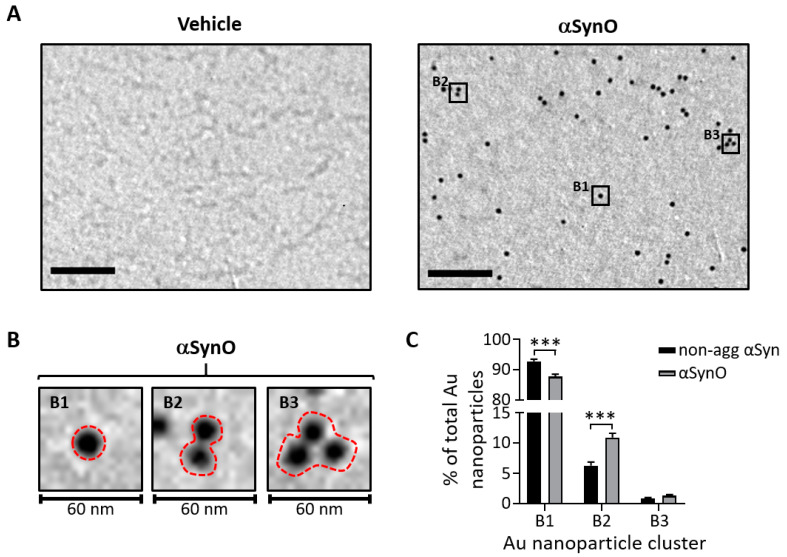
Ultrastructural analysis of αSyn species. (**A**) Representative TEM micrographs showing the vehicle (left) and αSynO (right), negatively stained with phosphotungstic acid and labeled using an anti-αSyn primary antibody. Immunoreactivity was detected using an Au nanoparticle (10 nm)-conjugated secondary antibody. The scale bar is 0.2 μm. (**B**) Magnified images of B1–B3 areas of αSyn electron micrograph in (**A**) showing the gold nanoparticle clusters: (B1) Au nanosphere monomers, (B2) Au nanosphere dimers, and (B3) Au nanosphere trimers. (**C**) Quantification of the number of Au nanoparticles per cluster of αSyn oligomers. Bars represent mean ± SEM (*n* = 4 independent experiments, *** *p* < 0.001; unpaired *t*-test).

The morphology of αSyn low-molecular-weight species was characterized by transmission electron microscopy (TEM) using the syn-211 antibody. No evidence of αSyn fibrils was found in the αSynO solution (Figure 2A), in contrast to what was observed for oligomerized Aβ_40_ (Supplementary Figure S1). We found three types of clusters of Au nanoparticles in the αSynO samples, which were defined as (B1) monomers, (B2) dimers, and (B3) trimers (Figure 2B). TEM quantification revealed that although the majority of the αSyn solution is composed of monomer particles (Figure 2C) for both non-agg αSyn (~93%) and αSynO (~88%), the number of monomers was significantly lower in the αSynO sample compared to the non-agg αSyn. The decrease in the number of monomers in the αSynO sample was accompanied by an increase in the number of dimers (~11%), which was significantly higher compared to the non-agg αSyn. No significant difference was observed in the number of trimers for both conditions. These findings were in line with the silver-staining and Western blot results.

In summary, the results indicated that under these oligomerization conditions, we obtained low-molecular-weight species of αSyn that predominantly assemble into dimers. These results agree with a previous study that showed that these low-molecular-weight diffusible species bind to neuronal membranes, producing ion-conducting pores, increasing intracellular calcium, and subsequently enhancing synaptic transmission [24]. Furthermore, confocal microscopy analysis of the αSyn-associated puncta to the neurons showed a puncta appearance without the presence of large aggregates.

### 2.2. Intracellular αSyn Oligomers Increased Synaptic Transmission In Vitro

**Figure 3:**
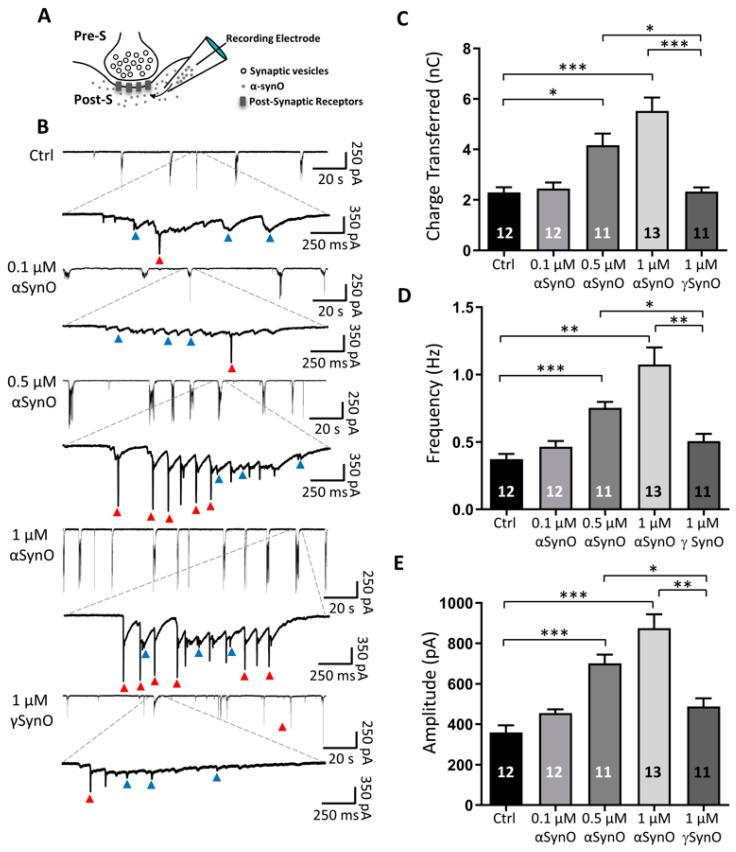
Intracellular αSyn oligomers increase synaptic transmission in a dose-dependent manner. (**A**) Schematic representation of postsynaptic recordings, showing the pre-synaptic (Pre-S) and postsynaptic (Post-S) compartments, and the intracellular dialysis of the recorded neuron with αSynO, using the patch-clamp electrode. (**B**) Representative recordings of postsynaptic currents from hippocampal neurons that were intracellularly dialyzed with a solution containing intracellular solution (Ctrl), αSynO (0.1, 0.5 and 1 µM) or 1 µM γSynO (holding potential (Vh) = −60 mV). The traces showed a rapid and marked increase in the bursts of synaptic currents (blue arrowheads) and spikes in the current recording mode (red arrowheads), as the concentration of αSynO was increased. (**C**–**E**) Quantification of charge transferred (fC), frequency (Hz), and amplitude (pA) of synaptic currents in terms of the different conditions described in (**B**). Bars represent mean ± SEM (numbers in bars are neurons; * *p* < 0.05, ** *p* < 0.01; *** *p* < 0.001; one-way Welch’s ANOVA with Dunnett’s T3 post hoc test).

Oligomeric αSyn conformation has been reported to exert neuronal toxicity, leading to neuronal dysfunction and neuron loss [15,24]. In this study, we investigated the intracellular effect of αSynO on synaptic transmission. Accordingly, αSynO was dialyzed into primary hippocampal neurons via a patch electrode, during the recording of spontaneous postsynaptic currents (sPSC) (Figure 3A). Control synaptic current recordings were made using an intracellular solution containing the vehicle for αSynO (Supplementary Figure S2). The data showed spontaneous synaptic events (Figure 3B, blue arrowheads) with some of the total activity being mediated by spikes in the current recording mode (Figure 3B, red arrowheads). Applying 0.1 μM αSynO did not have a significant effect on the total synaptic transmission, while the intracellular application of 0.5 and 1 μM αSynO increased the presence of spontaneous synaptic events and spikes in the current recording mode in all neurons (Figure 3B). We calculated the charge transferred for each condition and found a significant increase in this parameter when 0.5 and 1 μM αSynO was present in the intracellular compartment (in nC; Control: 2.29 ± 0.20, 0.1 μM αSynO: 2.44 ± 0.24, 0.5 μM αSynO: 4.16 ± 0.46, 1 μM αSynO: 5.52 ± 0.53, Figure 3B,C). The intracellular dialysis of 1 μM γSynO did not affect synaptic transmission or the charge transferred (1 μM γSynO: 2.32 ± 0.16, Figure 3B,C). Similarly, 0.5 and 1 µM of αSynO increased the frequency of the recorded events (in Hz; control: 0.37 ± 0.03, 0.1 μM αSynO: 0.46 ± 0.04, 0.5 μM αSynO: 0.75 ± 0.04, 1 μM αSynO: 1.07 ± 0.12, 1 μM γSynO: 0.50 ± 0.05 (Figure 3D)). Finally, we also observed an increase in the amplitude of sPSC at 0.5 and 1 µM αSynO, while 0.1 µM αSynO and 1 μM γSynO did not demonstrate significant differences when compared with control conditions (in pA; control: 359 ± 34.9, 0.1 μM αSynO: 454 ± 19.0, 0.5 μM αSynO: 701 ± 43.5, 1 μM αSynO: 875 ± 68.6, 1 μM γSynO: 488 ± 40.2 (Figure 3E)).

**Figure 4:**
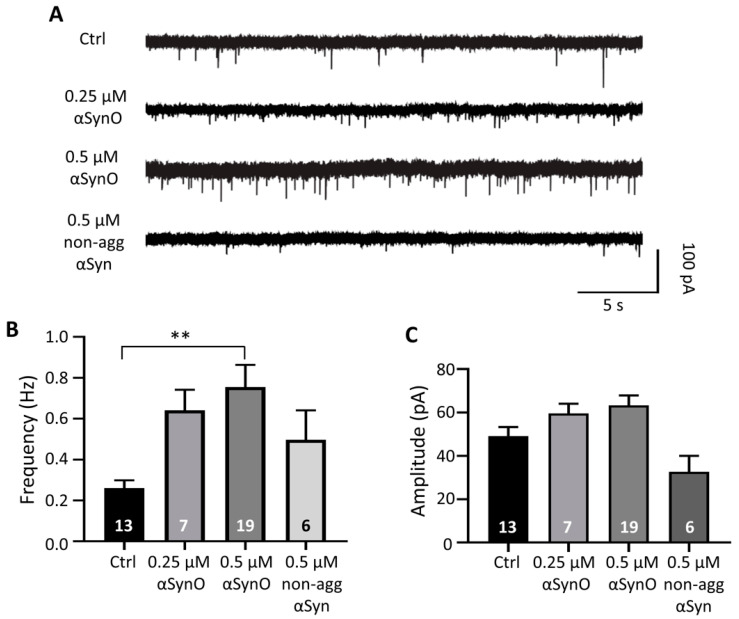
Intracellular αSynO increases the miniature postsynaptic currents. (**A**) Representative recordings of miniature postsynaptic currents (mPSCs; 0.1 µM TTX) from control and intracellularly dialyzed hippocampal neurons, with a solution containing αSynO (0.25 and 0.5 µM) or 0.5 µM non-agg αSyn (holding potential (Vh) = −60 mV). (**B**,**C**) Graphs show the effect on the frequency (Hz) from the different conditions described in (**A**). No significant changes in amplitude were found. Bars represent mean ± SEM obtained from the indicated number of neurons (** *p* < 0.01; one-way ANOVA with Bonferroni post hoc test).

Moreover, we measured the effect of αSynO on miniature PSC (mPSCs). The mPSCs were recorded after potential blocking action with tetrodotoxin (TTX; 0.1 μM; Figure 4A). Our results showed an increase in the mPSCs frequency with 0.25 μM αSynO (control: 0.26 ± 0.04 Hz vs. 0.25 μM αSynO: 0.64 ± 0.10 Hz); however, this effect was not significant (Figure 4B). On the other hand, when cells were dialyzed with 0.5 μM αSynO, there was a significant increase in the mPSCs frequency (control: 0.26 ± 0.04 Hz vs. 0.76 ± 0.11 Hz; Figure 4B). Conversely, no significant difference in mPSCs frequency was observed between hippocampal neurons dialyzed with non-aggregated αSyn, compared to the control group (control: 0.26 ± 0.04 Hz vs. 0.49 ± 0.14 Hz; Figure 4B). Although the frequency of mPSCs significantly increased with 0.5 μM αSynO, there was no significant difference in the amplitude of mPSCs (control: 49.2 ± 4.12 pA vs. 0.5 μM αSynO: 63.4 ± 4.53 pA, Figure 4C). This result suggests that the increase in synaptic transmission caused by intracellular αSynO involves a presynaptic effect.

### 2.3. In Silico Characterization of the Interaction between αSyn and M30

We recently reported the use of a small molecule having neuroprotective properties against Aβ toxic effects. This molecule, referred to as M30, inhibited aggregation by interacting with the C-terminal portion of the Aβ monomer, a highly hydrophobic region of the peptide. Remarkably, this hydrophobic domain or pocket has also been described for other amyloidogenic proteins, such as αSyn, and is easily targeted by small molecules such as M30 [26]. Thus, we performed a docking analysis, followed by molecular dynamics simulations (MD), to evaluate the interaction between αSyn and M30.

**Figure 5:**
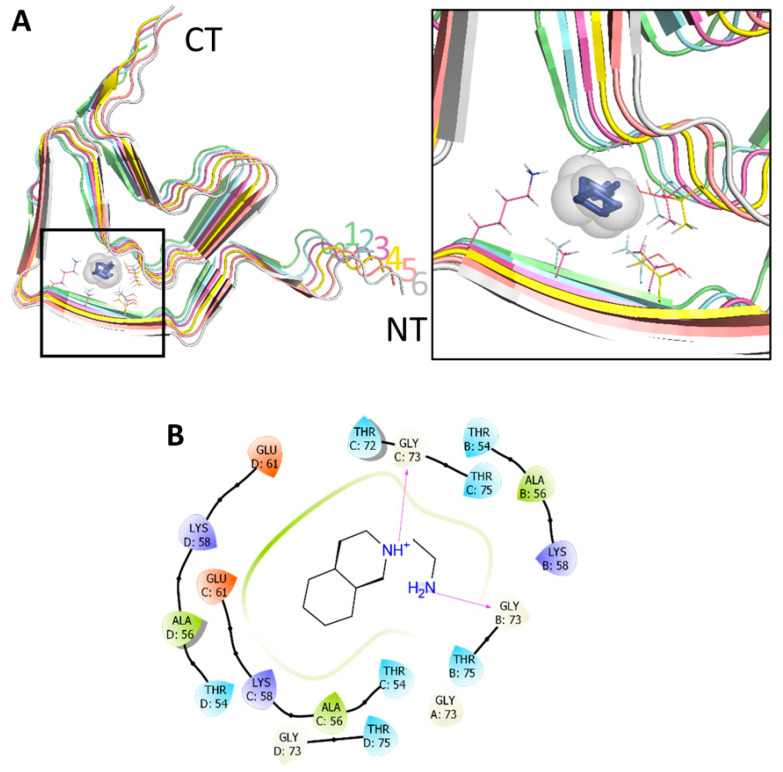
Interaction of M30 with αSyn fibers, predicted by docking. (**A**) Representative complex between M30 and αSyn fibril, composed of 6 identical subunits. The side-chains of amino acids close to M30 (cutoff 4Å) are shown. The right panel shows a magnification of the interaction region. (**B**) Schematic representation of the binding site, with amino acids colored according to their properties: red (negatively charged), blue (positively charged), green (hydrophobic), cyan (polar), gray (solvent exposure). The magenta arrow represents H-bonds.

The high-resolution cryo-EM structure of an αSyn fibril (PDB ID: 6A6B), as used in all predictions, is composed of six subunits and each of them is constituted by seven β-strands that are connected by six turns [27]. The first step in evaluating the ability of M30 to interact with the αSyn fiber was to create complexes using protein-ligand docking. The M30 molecule was able to strongly interact with the fiber, showing a binding affinity of −5.7 kcal/mol, as reported by AutoDock Vina (Figure 5A). When the ΔGbind was calculated, we obtained a value of −40.61 kcal/mol that confirmed an energetically favorable interaction. A more detailed analysis of the binding site showed the presence of two important regions between the amino acids 54TVAEKTKE61 and 72TGVT75, highlighting the formation of 2 hydrogen bonds with the glycine amino acids located on the B and C chains (B:73, C:73), in a mainly hydrophilic environment (Figure 5B). Likewise, alternative positions for M30 were detected in the fiber that correspond to the same binding sites formed by the adjacent subunits, in an arrangement that resembles a tunnel with M30 inside it (Supplementary Figure S3).

**Figure 6:**
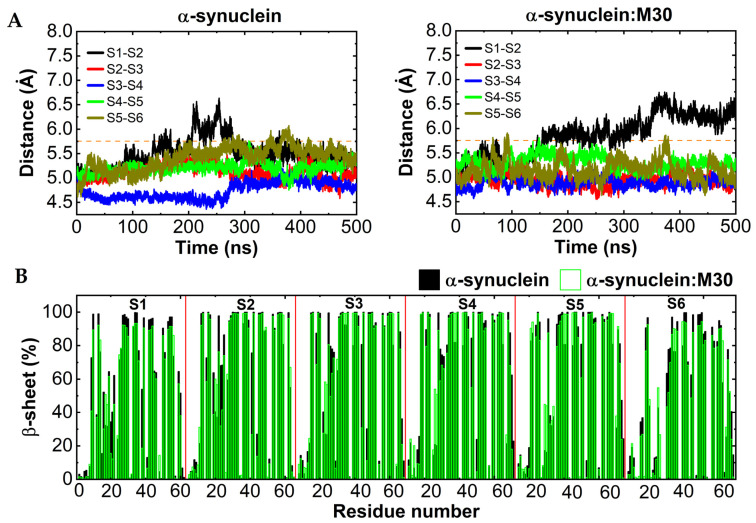
M30 induces changes in αSyn fibrils, as predicted by molecular dynamics. (**A**) The variation of the averaged distance between neighboring subunits over the five trajectories with respect to the time series, in the case of αSyn and αSyn:M30. (**B**) Secondary structure per residue, calculated by averaging over all αSyn and αSyn:M30 conformations acquired between 200 and 500 ns of each trajectory. Subunits are labeled in the diagram.

To elucidate the impact of the M30 interaction on a reported αSyn structure (hexamer), we examined the structural characteristics of the αSyn:M30 complex by using an extensive (2500 ns) MD simulation in an aqueous environment. Changes in the distances between neighbouring subunits in the αSyn fibril, upon M30 interaction during the simulation, were analysed (Figure 6A). In the case of the αSyn alone, the distances measured between neighbouring subunits are in the range of 4.5 to 5.7 Å during the MD simulation, which coincided reasonably well with the experimental data [27]. In the case of αSyn:M30, the distance between subunits 1 and 2 was significantly increased in the range of 5.7 to 6.7 Å after 150 ns; therefore, the S1 subunit showed dissociation from the fibril structure. A secondary structure analysis in the presence of M30 found that αSyn:M30 possessed lower beta-sheet and higher coil contents at each subunit, in comparison with the αSyn fibril (Supplementary Table S1). Also, significant structural changes were noticed in the S1 and S6 subunits with M30 (Figure 6B, Supplementary Figure S4). The resulting interaction with M30 can lead to a lower beta-sheet and higher coil content in the αSyn fibril. In addition, upon M30 interaction, the contacts between S1 and S2 subunits were reduced by increasing the strength of the aforesaid hydrophobic contacts in the S1 subunit (Supplementary Figure S5). In the case of αSyn:M30, the intra-hydrophobic contacts were found between β–β strands and the N-terminal region–β strands in the S1 subunit. These results agree with the longer distance observed between S1 and S2 subunits. In contrast, these interactions were missing in αSyn, and only the N-terminal region formed a contact with β at the S6 subunit (Supplementary Figure S5). It is noteworthy that the inter-and intra-subunits salt bridges play a significant role in α-Synuclein fibril stability, which is affected by M30 interaction through decreasing inter-subunit salt bridges (Supplementary Figure S6).

### 2.4. M30 Inhibited αSyn Aggregation

**Figure 7:**
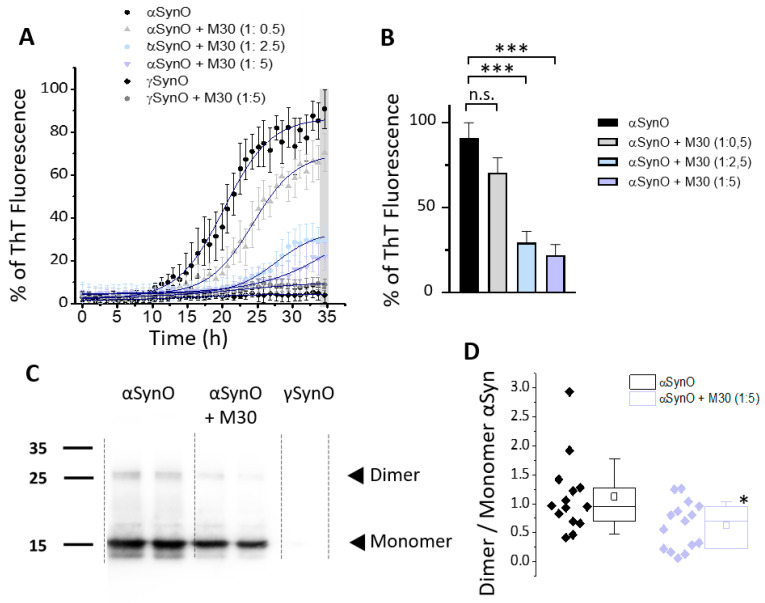
Effects of M30 on αSyn oligomerization. (**A**,**B**) ThT fluorescence assay to measure the β-sheet content in different samples of αSyn, co-incubated in the presence of M30 (ratios αSyn:M30 used: 1:0.5, 1:2.5 and 1:5). Briefly, 24 µM ThT in DPBS was used at pH 4.0 at 37 °C for 35 h, with fluorescence readings at 485 nm to monitor αSyn aggregation. The graph also shows ThT fluorescence, adjusted to a Boltzmann sigmoidal curve (navy blue lines). Values are expressed as a percentage of the maximum ThT fluorescence. The graph represents the mean ± SEM (*n* = 4 independent experiments). The gray box at the end of the recording period indicates the time at which the statistical comparison was performed and plotted as a bar graph (**B**), for αSyn:M30 ratios of 1:2.5 and 1:5. Bars represent mean ± SEM (*** *p* < 0.001; one-way ANOVA with a Bonferroni post hoc test, n.s.: no significant). (**C**,**D**) Western blot analysis showed a decrease in the presence of dimers when the αSyn sample was co-incubated alone (black diamonds) or with M30 (purple diamonds, 1:5). No signal was observed for γSyn. As expected, the monomer:dimer αSyn ratio for the samples that contained M30 was significantly decreased. Boxes indicate interquartile range (IQR); center lines, median; whiskers, 1 × SD (*n* = 8 independent gels, * *p* < 0.05; Mann–Whitney U test).

Considering the previous “in silico” data, we tested whether the presence of M30 affects the aggregation propensity of αSyn. For this, we constructed a concentration-effect curve in the presence of an increasing concentration of M30, in the presence of a fixed concentration of αSyn. Thus, αSyn was co-incubated with M30 (αSyn:M30, ratio 1:5). The effect of M30 on αSyn aggregation was studied using a ThT fluorescence assay (Figure 7A). We found that M30 was able to reduce the β-sheet content when the ratio αSyn:M30 was 1:5 and 1:2.5 (no significant differences were found for the ratio 1:0.5) The statistical comparison was performed at the end of the experiment (Figure 7A, indicated by the gray box) and plotted as a bar graph for each condition with αSyn. Statistical differences were found for αSyn:M30 (1:2.5) and αSyn:M30 (1:5), when compared to αSyn without M30 co-incubation (Figure 7B). In addition to the ThT assay, we confirmed the effects of M30 on αSyn aggregation by Western blot and found a reduction in the formation of dimers (Figure 7C). In consequence, the dimer/monomer ratio in the sample with M30 also decreased (Figure 7C,D). As expected, no significant aggregation was observed for γSyn (Figure 7A–C).

### 2.5. The Increase in Synaptic Transmission Induced by Intracellular αSynO Was Reduced by M30

**Figure 8:**
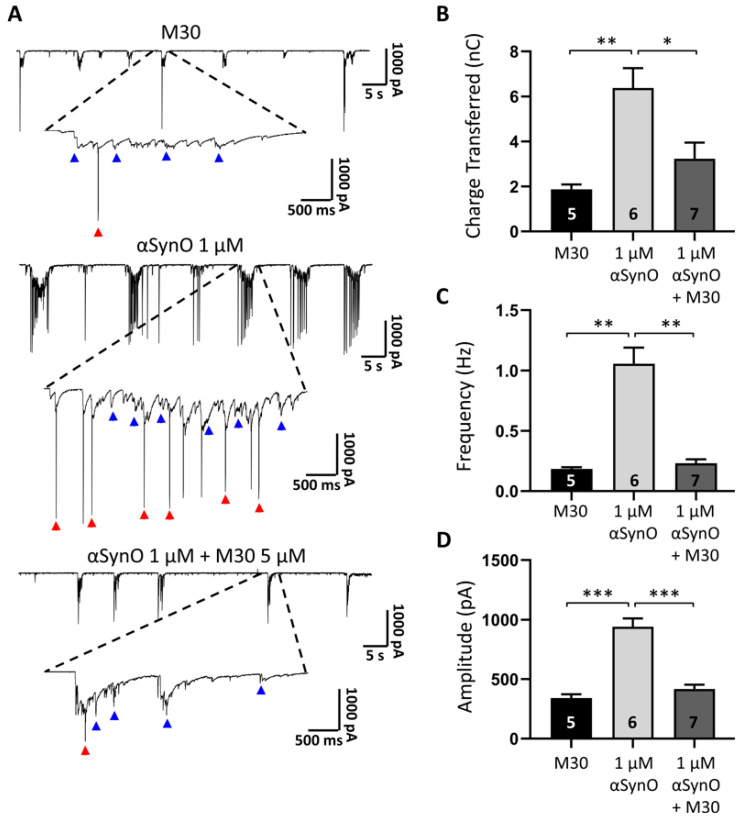
The intracellular effects of αSynO are diminished in the presence of M30. (**A**) Postsynaptic current recordings of primary hippocampal neurons were intracellularly recorded with a solution containing 1 μM of αSynO, in the absence and presence of 5 μM M30, for 24 h. Control condition shows the presence of spontaneous synaptic currents (arrows in blue) along with spikes in the current recording mode (arrows in red) and both currents are increased when αSynO 1 µM is applied intracellularly through the recording electrode. Oligomerization in the presence of M30 decreases this effect. (**B**–**D**) Quantification of charge transferred (fC), frequency (Hz), and amplitude (pA) from the conditions described in (**A**). Bars represent the mean ± SEM from the indicated number of neurons (* *p* < 0.05, ** *p* < 0.01; *** *p* < 0.001; one-way Welch’s ANOVA with Dunnett’s T3 post hoc test).

Consequently, our experimental strategy involved the study of sPSCs to assess whether M30 attenuated the effect of intracellular αSynO on synaptic activity. The recordings showed an important increase in spontaneous bursts of synaptic currents when intracellular αSynO was present (Figure 8A, blue arrowheads). It is also possible to observe that part of the total activity is mediated by spikes in the current recording mode, which is also augmented in the presence of 1 μM αSynO (Figure 8A, red arrowheads). Furthermore, statistical analysis showed that intracellular αSynO significantly increased the charge transferred during the recording, compared to the control condition (in nC; M30: 1.87 ± 0.22 vs. 1 µM αSynO: 6.38 ± 0.87; Figure 8B) indicating that more current per unit of time was flowing through the membrane, compared to control conditions. However, a significant decrease was observed in sPSCs and the charge transferred when αSynO was co-applied with M30, reaching values statistically similar to that of control (in nC; control: 1.87 ± 0.22 vs. 1 µM αSynO + M30: 3.23 ± 0.71; Figure 8B). Similarly, the presence of M30 reduced the effect of intracellular αSynO on the frequency (in Hz; M30: 0.14 ± 0.22, 1 µM αSynO: 1.05 ± 0.13, 1 µM αSynO + M30: 0.23 ± 0.03; Figure 8C) and amplitude of recorded sPSCs (in pA; M30: 340 ± 32.2, 1 µM αSynO: 940 ± 70.1, 1 µM αSynO + M30: 471 ± 36.4; Figure 8D).

## 3. Discussion

### 3.1. Intracellular Effects of αSynO in Synaptic Physiology

A significant number of studies have described the presence of αSynO in the intracellular compartment, including nuclear fractions, mitochondria, lysosomes and exosomes [28,29,30]. Additionally, the presence of αSyn has been reported in axons [31] and pre-synaptic terminals [32,33,34,35,36,37], suggesting that this accumulation is important for the onset of PD synaptopathy [38]. In addition, several mechanisms for the intracellular uptake of αSyn have been reported, including passive diffusion through the plasma membrane [39,40], endocytosis [2,40,41,42], and the exosome-mediated transfer of αSyn into a recipient cell [28]. Actually, it is believed that the toxic actions of α-syn arise from the accumulation of diverse molecular species, defining a family of neurological disorders termed α-Synucleinopathies. The α-syn monomers can oligomerize, forming diverse soluble species that are able to alter membrane conductance when applied in a dissolved form in physiological solutions [24,43]. In the present study and in agreement with a previous study [24], we only detected low-molecular-weight oligomers. This was accompanied by a lack of evidence for αSyn fibrils when examined by electron microscopy.

Although the precise mechanism for toxic effects remains to be elucidated, this has been suggested as a possible pathway for the cell-to-cell spread of the pathology throughout the brain [44,45], similar to Aβ and prion (PrP) amyloidopathies [46,47]. The present data is particularly interesting because we found that even though αSyn was applied in the postsynaptic domain, it was able to alter the presynaptic release machinery, thus increasing the frequency of miniature currents. Although we do not have an explanation for this effect, it agrees with the notion that αSyn can propagate trans-synaptically. For example, recent studies have shown that αSyn accumulated and impaired the interconnection of the sensory neuron circuitries related to pain transmission [48,49]. In addition, the oligomerization of αSyn from monomers to fibrils depends on intracellular conditions and it is likely that all species present toxicity, although at different potencies [50,51]. This supports the strong effect produced by the predominantly low-molecular-weight species in the current αSyn preparation, which are able to diffuse into the intracellular milieu.

Now, the question that remains to be answered is: what is the contribution of this intracellular accumulation to synaptic function? In the present study, and using the patch electrode to deliver the peptide, we found that 0.5 µM of αSynO was enough to drastically increase the frequency and amplitude of postsynaptic currents by ~50%, as well as the total charge transferred during these recordings, but no significant effects were observed for γSyn. This effect was concentration-dependent, since it was enhanced when the concentration was doubled within the patch electrode (1 µM Syn). Interestingly, using the same experimental approach to deliver the peptide intracellularly, Wu et al. showed that αSyn decreased the frequency of miniature excitatory postsynaptic currents (mEPSC) without affecting amplitude. In addition, the intracellular presence of these aggregates was enough to decrease synapse formation by altering the dendritic spine dynamics [15]. Although we did not measure inhibitory and excitatory currents separately, the increase in the frequency of sPSC and mPSC, which contrasts with the study by Wu et al., might be explained by the fact that they used a high concentration of αSyn “fiber-enriched” preparations (5 µM), contrary to our solution that lacked fibers (1 µM).

Our results also suggest that changes in neuronal excitability might be occurring during the postsynaptic recordings, as evidenced by the higher number of recorded voltage-clamped spikes as the concentration of αSynO increased. In fact, the effects described for intracellular αSynO regarding the frequency, amplitude, charge transferred, and increased number of voltage-clamped recorded spikes in the recordings are remarkably similar to those described for intracellular Aβ oligomers (Aβo) in a previous study by our laboratory [52]. Contrary to our findings, Yamamoto et al. reported that the intracellular diffusion of a solution containing stable higher-order αSynO (larger than 100 kDa) decreased neuronal excitability [16]. Moreover, this effect was triggered by intracellular calcium release, which was mediated by IP3R. Although the concentrations used were very similar to those in our study (1 µM), the αSynO solutions were obtained after co-incubation of αSyn with dopamine for 3 days, a quite different method from the one used in our investigation. In fact, two additional reports also show that the intracellular dialysis of αSyn containing high molecular-weight oligomers reduced pyramidal cell excitability, both in primary hippocampal neurons [15] and in neocortical slices from mice [17]. Since all these studies used higher-molecular-weight conformers (>100 kDa) intracellularly, the differences found in our study in terms of excitability could be due to the fact that dimers were the most abundant species in our preparations (~35 kDa). However, this clearly shows that these intracellular species are neuroactive and have a direct effect on neuronal physiology by affecting excitability, leading to synaptic alterations and neurodegeneration [17]. Further studies are needed to clarify the differential effect for high- and low-molecular-weight αSynO.

Although we do not describe a particular mechanism by which αSynO might affect neuronal physiology, another study using inside-out single-channel recordings showed that the application of 50–100 nM αSynO to the inner leaflet of the bilayer increased the conductance of the membrane [53]. Although the type of species present in the recording solution was not described or characterized in detail, the author attributed this effect to the formation of αSyn “channels” in the inner leaflet of the bilayer that were responsible for causing a disruption in ionic homeostasis in the neurons, in the same manner as externally applied αSynO, which significantly increased neuronal excitability [53]. These “channels” resemble the pore-forming actions described by our and other laboratories for Aβ and αSyn oligomers, i.e., the disruption of the lipid bilayer, leading to synaptic failure [24,54,55,56,57].

Additionally, it has also been previously identified that αSyn interacts and regulates the size of the presynaptic vesicle pool in the brain and modulates synaptic transmission [35,58,59]. Intracellular αSyn has also been shown to influence the intracellular dynamics affecting axonal transport, leading to a decreased distal vesicle pool and a reduction in neurotransmitter release [60]. However, the key event(s) that trigger the transition from increased synaptic function to synaptic dysregulation has yet to be identified, but it is tempting to hypothesize that a sustained increase in neurotransmission could cause vesicle pool depletion [61], as previously described for other well-known amyloid peptides, such as Aβ [62,63].

### 3.2. Neuroprotective Properties of M30 against αSynO-Mediated Synaptic Effects

Finally, using combined in silico and in vitro techniques, we evaluated if a previously identified small molecule with neuroprotective properties (M30) could diminish or ameliorate the observed effects on synaptic physiology. M30 was previously reported by our laboratory as a peptide-based research molecule, derived from a massive screening, one that was previously shown to interfere with myriad toxic-induced Aβ effects [22]. At the first instance, docking analysis predicted an energetically favorable interaction for M30 and αSyn in the NAC domain (residues 61–95) where the structure folds itself, creating a hydrophobic cavity. The NAC domain is particularly important for the aggregation of αSyn [64,65,66] and actually initiates the early assembly of αSyn in the oligomerization process [67], contributing to protofilament stability [68]. Moreover, it was possible to identify key segments, such as 54TVAEKTKE61 and 72TGVT75A56, that might interact with M30. The results nicely correlate with the lower beta-sheet and higher coil contents observed in MD simulations. Together with the WB analysis and the ThT fluorescence binding assay that validated the decrease in β-sheet content, the study showed that M30 can interfere with the oligomerization process. In silico data also showed strong evidence that M30 can enhance the dispersion of the AS fibril structure. Thus, it is possible that the effects of M30 on αSyn aggregation could also block the abovementioned effect of oligomer-mediated membrane perforation, which has already been described elsewhere for Aβ oligomers [22]. This makes M30 a molecule with unique characteristics and significant therapeutic potential since it would be active for myriad Aβ- and αSyn-mediated toxic effects including its aggregation and propagation, which are central steps in its neurotoxic pathway.

In summary, we report a rapid and potent synaptic effect of αSynO that was ameliorated by co-incubation with a previously described small molecule (M30) that interferes with the oligomerization process, resulting in the formation of structurally distinct aggregates. Given the fact that the presence of these species has been shown to be important in the etiology of different synucleopathies, this study provides new and key information regarding the physiological consequences of intracellular αSynO accumulation, as well as the identification of a molecule capable of modifying the structural properties of αSyn to reduce the toxic actions of these oligomers. Thus, having a better understanding of the intracellular mechanisms of αSyn pathology and finding neuroactive molecules that interfere with its toxicity may be important for generating new disease-modifying therapies for common neurodegenerative diseases like PD and DLB [1,69].

## 4. Materials and Methods

### 4.1. In Vitro αSyn Oligomer Preparation

The methodologies for oligomer preparation and Western blot characterization have been previously described [24]. For instance, recombinant human αSyn (1–140), purchased from rPeptide (Athens, GA, USA), was resuspended in sterile water to give a final concentration of 346 µM. Aliquots were kept at −20 °C. The αSyn oligomerization was conducted by diluting the aliquots in sterile Dulbecco’s phosphate-buffered saline (DPBS) (Thermo Fisher Scientific Inc., Waltham, MA, USA) adjusted to pH 4.0 (soluble protein stock of 13.3 or 60 µM, as required), followed by incubation at 37 °C, with constant vertical stirring at 800 rpm for 24 h in a Thermomixer Compact (Eppendorf AG, Germany). After the oligomerization process, the samples were centrifuged at 10,621× *g* for 15 min. The electrophoresis experiments were performed with the supernatant (soluble fraction). Samples were not boiled. The γ-synuclein (γSyn) was used as a control for aggregation because, in this experimental condition, it does not exhibit a high propensity for aggregation when compared with αSyn [70].

### 4.2. In Vitro Aβ Oligomers Preparation

Aβ oligomers were prepared as previously described [71]. Briefly, Aβ was dissolved in 1,1,1,3,3,3-Hexafluoro-2-propanol (HFIP) (10 mg/mL) (Merck Millipore, Burlington, MA, USA) and incubated in a parafilm sealed tube at 37 °C for 2 h. Then, the solution was incubated at 4 °C for 20 min and aliquots of 5 µL were placed in 1.5 mL open-lid Eppendorf tubes to allow evaporation (Eppendorf AG, Hamburg, Germany). Aliquots were stored at −20 °C. To obtain an oligomer-rich solution, nanopure water was added to obtain a final concentration of 80 µM and the tubes were incubated at room temperature for 20 min. Subsequently, a Teflon-coated magnetic stir bar was added to the solution (size: 2 × 5 mm) and stirred at room temperature (typically 21 °C) at 500 rpm for 24 h.

### 4.3. M30 Molecule Preparation

M30 (M30 2-octahydroisoquinolin-2(1H)-ylethanamine) was prepared as previously described [22]. Briefly, M30 (Matrix Scientific, Columbia, SC, USA) was dissolved in dimethyl sulfoxide (DMSO) to a final concentration of 10 mM, and aliquots were stored at −20 °C until use. For a lower stock concentration, M30 was dissolved in ultrapure water or saline.

### 4.4. Aggregation Studies Using Thioflavin T Fluorescence

Briefly, αSyn (32 µM) was aggregated in the presence of 24 µM thioflavin T (ThT) at pH 4.0 in DPBS. Amyloid beta 1–40 (Aβ_40_; GenicBio, Shangai, China) in PBS (phosphate-buffered saline, (Gibco, Waltham, MA, USA), PBS pH 7.4 and DPBS pH 4.0, in the presence of 24 µM ThT, were used as controls. The resulting fluorescence was measured with a NOVOstar (BMG LABTECH, Ortenberg, Germany) with the excitation filter set at 485 nm and the emission filter at 520 nm. The fluorescence signal was fitted to Boltzmann-sigmoidal kinetics (1):

(1) $$y=\frac{A1-A2}{1+e^{\left(x-x_{0}\right)/dx}}+A2$$

where *A*1 and *A*2 are the initial and maximum fluorescence, respectively, *dx* is the rate constant, and *x*_0_ is the time at half-maximum fluorescence.

### 4.5. Silver-Staining

Equal amounts of recombinant human αSyn (1–140) were separated on 15% SDS polyacrylamide gels. Gels were silver-stained using a Silver SNAP Stain II^®®^ kit (Bio-Rad, Hercules, CA, USA.) according to the manufacturer’s instructions. Briefly, the gels were washed several times in 30% ethanol/10% acetic acid in sterile water, immediately following electrophoresis. The gels were then transferred to a sensitizer^®®^ solution. Next, the gel was placed in stain solution for 30 min and rinsed with developer solution. The reaction was stopped by washing the gel with 5% acetic acid.

### 4.6. Western Blot

Equal amounts of recombinant αSyn were loaded onto Tris-tricine gels, separated electrophoretically, and transferred onto nitrocellulose membranes, blocked with 5% non-fat milk in Tris buffered saline with tween 20 (TBS-T)for 1 h at room temperature, then incubated for 1 h with anti-αSyn (211) antibody (1:1000, mouse, Santa Cruz Biotechnology, Santa Cruz, CA, USA) at room temperature. After washes with 1X TBS and 0.1% Tween 20, membranes were incubated for 2 h with anti-mouse secondary antibodies conjugated to horse-radish peroxidase (1:5000, Santa Cruz, CA, USA). Immunodetection was performed using the western lightning ECL kit (PerkinElmer, Waltham, MA, USA). Band intensities were analyzed and compared using ‘ImageJ’ 1.8.0_112 (NIH, https://imagej.nih.gov/ij, accessed on 17 October 2021).

### 4.7. Transmission Electron Microscopy

An aliquot from control (DPBS, pH 4), non-aggregated and oligomerized αSyn, and oligomerized Aβ_40_ were adsorbed onto Formvar-coated nickel grids for 5 min. Next, the grids were blocked with 3% bovine serum albumin (BSA) in Tris-HCl (pH 7.4) to reduce the nonspecific background. The grids were then incubated with primary antibodies (monoclonal anti-αSyn 211, dilution 1:50, or anti-Aβ, dilution 1:50) in 0.1% BSA for 30 min. Next, the grids were washed in TBS (pH 7.4), incubated with gold (Au) nanoparticle (10 nm)-conjugated anti-rabbit IgG secondary antibody (Kenilworth, NJ, Merck), and then stained with 5 μL of 0.2% (wt/vol) phosphotungstic acid. Samples were air-dried and examined using a JEOL 1200 EX II electronic microscope (Jeol, Tokyo, Japan). Images were subjected to quantification using ImageJ. Data collected from Au nanoparticle size measurements were used to create a nanoparticle size to define Au nanoparticle clusters. Au nanoparticles that were smaller than 350 nm^2^ were denominated as monomers, Au nanoparticles between 350 and 800 nm^2^ were denominated as dimers, and Au nanoparticles larger than 800 nm^2^ and smaller than 2000 nm^2^ were denominated as trimers.

### 4.8. Primary Culture of Hippocampal Neurons

Hippocampal neurons were obtained from C57BL/J6 mice or Sprague Dawley rat embryos, as previously described [72]. Animal care and protocols were in accordance with the National Institutes of Health (NIH) recommendations and approved by the Ethics Committee at the University of Concepcion (Review # 1189752, 15 April 2018). Briefly, hippocampal tissue was harvested from 18- to 19-day-old embryos. The pregnant animal was anesthetized with isoflurane and subsequently euthanized by cervical dislocation. The embryos were removed and rapidly decapitated. The brains were removed, and the hippocampi were dissected from the cortices free of meninges. The hippocampus was mechanically and enzymatically dissociated. Hippocampal cells were seeded on poly-L-lysine (0.25%; Sigma, St. Louis, MI, USA)-coated plates at a density of 32 × 10^4^ cells/mL. The cultures were maintained in a feeding medium consisting of 90% minimal essential medium (MEM, GIBCO, USA), 5% heat-inactivated horse serum (HyClone, USA), 5% fetal bovine serum (Life Technologies), and a mixture of nutrient supplements.

### 4.9. Whole-Cell Patch-Clamp Recording

For voltage-clamp experiments in whole-cell mode, the dish culture medium was replaced with a normal external solution (NES) containing (in mM): 150 NaCl, 5.4 KCl, 2.0 CaCl_2_, 1.0 MgCl_2_, 10 glucose and 10 HEPES (pH 7.4, adjusted with NaOH, 310 mOsm/L). Cells were stabilized at room temperature for 20 min before beginning the experiments. The internal solution used to record spontaneous postsynaptic currents (sPSCs) contained (in mM): 120 KCl, 2.0 MgCl_2_, 2 Na_2_ATP, 10 BAPTA, 0.5 NaGTP and 10 HEPES (pH 7.4 adjusted with KOH, 290 mOsm/L). The membrane currents in single neurons were recorded by adjusting the membrane potential to −60 mV with an Axopatch-200 B amplifier (Molecular Devices, San Jose, CA, USA) and under visualization with an inverted microscope (Nikon Eclipse TE200-U, Tokyo, Japan). The acquisition was made using a computer connected to the recording system, using a Digidata 1440A acquisition card (Molecular Devices, San Jose, CA, USA) and the pClamp10 software (Molecular Devices, USA). Electrodes with a resistance of 4–5 MΩ were pulled from borosilicate capillaries (WPI, Sarasota, FL, USA) in a horizontal puller (P1000, Sutter Instruments, Novato, CA, USA). A 5 mV pulse was used to monitor series resistance throughout the recording period and only cells with a stable access resistance (less than 20 MΩ and that did not change more than 20%) were included for data analysis. To study miniature postsynaptic currents (mPSCs), 100 nM tetrodotoxin (TTX; Alomone Labs, Jerusalem, Israel) was applied in the NES of the well containing the cells.

### 4.10. Docking Analysis

A docking protein-ligand analysis was performed using the structure of the αSyn fiber obtained from the Protein DataBank (PDB ID: 6A6B) [27]. M30 comes from a virtual screening for the beta-amyloid peptide [22] and its structure was obtained from the ZINC15 database (ZINC38790891). Before docking simulations, the αSyn structure was prepared so as to incorporate hydrogens, assign bond orders, fill in missing side-chains, and generate protonation states at pH 7 ± 0.2. All complexes were created by AutoDock Vina [73], using a grid that encompassed 6 identical fiber subunits. Analysis of the interface αSyn:M30 included structural and energetic parameters and was performed with the same software. Finally, to predict the ligand-binding affinity, a theoretical ΔG_bind_ was calculated via an energy calculation, MM-GBSA, using Prime (Schrödinger, LLC, New York, NY, USA, 2016). All images presented were created using PyMol (version 1.5, DeLano Scientific LLC, Palo Alto, CA, USA).

### 4.11. Molecular Dynamics Simulations

The M30 molecular geometry was optimized using Gaussian09 with the Hatree-Fock/6-31G* level of theory, and the parameters of the ligand were obtained from the generalized amber forcefield through the Antechamber tool, using partial charges from a quantum chemical calculation based on the restrained electrostatic potential (RESP). Five replicas were generated for the αSyn and the complex αSyn:M30. Both conditions were separately solvated in an explicit 20,235 and 20,231 TIP3P water, in a cubic box with a total volume equal to 793,949 Å3. The system was neutralized by adding the required Cl^−^ ions. The system energy was minimized in successive steps: 500 steps of the steepest descent method, 500 steps of the conjugate gradient method with a restrained complex, using a harmonic potential with spring contacts equal to 500 kcal/mol/Å2, 1000 steps of the steepest descent, and 1500 steps of the conjugate gradient minimization method without restraint. The system was equilibrated in two steps after the energy minimization scheme: first, an NVT (constant temperature) ensemble was applied for 20 ps and the temperature was gradually increased from 0 to 300 K; second, the NPT (constant pressure) ensemble was employed for 200 ps to achieve the correct water density at 1 atm pressure. Each replica was subjected to 500 ns production run in the NPT ensemble. Production data were collected every 20 ps. All bond lengths were constrained by the SHAKE algorithm. The particle mesh Ewald (PME) method was used for the long-range electrostatic interactions, and a typical 10 Å cutoff range was fixed to calculate the electrostatic interactions. A Langevin thermostat was used to control the temperature. A simulation of 2500 ns (5 replicas × 500 ns) was carried out for the αSyn and αSyn:M30 complexes. Molecular dynamics (MD) simulations were performed using the AMBER16 package [74,75].

### 4.12. Structural Analysis

We examined the structural stability of the fibril models with and without interaction with the M30 molecule, by studying the following analyses. The contact map was defined as two residues in contact once the distance between the centers of masses of any atom in those residues was below 6.5 Å; a secondary structure per residue of the fibril was calculated using the DSSP program [76,77]. The average secondary structure per residue was calculated by averaging over all the αSyn and αSyn:M30 fibril conformations collected between 200 and 500 ns of each trajectory; the distances between neighboring subunits in the fibril structure were computed using the “distance” program in Amber [78]; hydrogen bonds were considered when the X–Y distance in X–H … Y is smaller than 3.5 Å and the X–H … Y angle is larger than 135°. We measured the inter-subunit hydrogen bonds (HB), using the following equation: inter-subunit HB = total number of HB−intra-subunit; salt bridges between two charged residues were measured when the distance between two specific atoms was below 4.5 Å. Salt bridges were determined using the following equation [79]:

(2) $$SB=\underset{i,j}{\sum }S_{i,\;j}$$

(3) $$S_{i,j}=1\;if\;r_{i,\;j}\leq 0$$

(4) $$S_{i,j}=0\;if\;r_{i,\;j}>0$$

(5) $$r_{i,j}=\left|r_{i}-r_{j}\right|-d_{0}$$

where *i* and *j* are running over different sets of atom pairs, each term of the pair being contained in a different portion of the system. Two sets of atoms were identified, one belonging to the positively charged group, N_ζ_(Lys), and the other to the negatively charged group, C_δ_(Glu). We inspected the contact as an inter- and intra-peptide salt bridge. *d*_0_ represents the distance between atoms *i* and *j*. The value of *d*_0_ was 4.5 Å.

### 4.13. Data Analysis and Statistics

The synaptic currents were analyzed as previously described [52]. Briefly, miniature postsynaptic current (mPSCs) frequency and amplitude were analyzed using the Mini analysis software (Synaptosoft, Inc., Leonia, NJ, USA). As a routine check, we visually inspected all events detected by the software and rejected any that did not exhibit the general expected form for synaptic events. For spontaneous postsynaptic currents (sPSCs), the area under the current trace was integrated and expressed as a charge transferred during the whole recording time, using Clampfit 10.5 (pClamp 11, Molecular Devices, USA). All data obtained from all parameters were plotted using Prism 6.0 (GraphPad, San Diego, CA, USA). Unless otherwise indicated, all data is shown as mean ± SEM for normally distributed populations, and as median and interquartile ranges (IQR) for non-normally distributed populations. Statistical analyses were performed using the two-tailed unpaired Student’s *t*-test (α = 0.05) or the two-tailed Mann–Whitney U test, as appropriate, after testing for normality with the Shapiro–Wilk test and for the homogeneity of variances with Levene’s test. A one-way ANOVA test (α = 0.05) was used to compare several populations of neurons, followed by an appropriate post hoc test. A probability level (*p*) < 0.05 was considered statistically significant (* *p* < 0.05, ** *p* < 0.01, *** *p* < 0.001).

## Data Availability

The datasets used and/or analyzed during the current study are available from the corresponding author on reasonable request.

## Supplementary Materials

The following are available online at https://www.mdpi.com/article/10.3390/ijms222413253/s1.

## Abbreviations

Aβ	Amyloid-β peptide
αSynO	αSynuclein oligomers
M30	2-Octahydroisoquinolin-2(1H)-ylethanamine
mPSC	Miniature postsynaptic currents
PD	Parkinson’s Disease
sPSC	Spontaneous postsynaptic currents
TTX	Tetrodotoxin
