# Supplementary material for: The Stimulatory Effects of Intracellular α-Synuclein on Synaptic Transmission Are Attenuated by 2-Octahydroisoquinolin-2(1H)-ylethanamine
Source: Int J Mol Sci. 2021 Dec 9;22(24):13253. doi: 10.3390/ijms222413253 (PMC8705949; doi:10.3390/ijms222413253)
Supplement: Supplementary file 1 [file ijms-22-13253-s001.zip › Supplementary Figures.pdf]

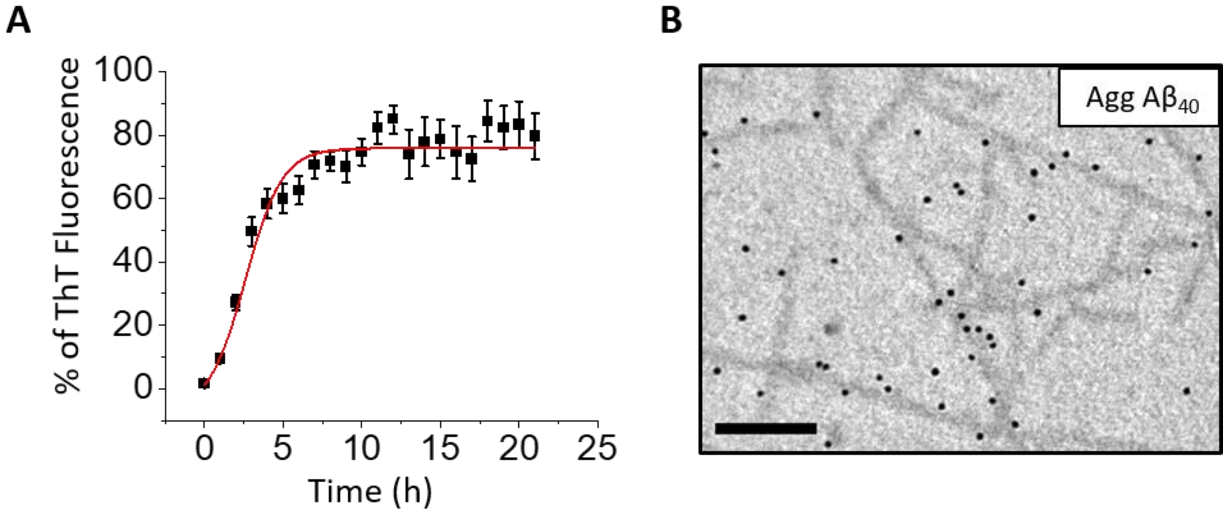

Supplementary Figure S1. Characterization of $A\beta_{1-40}$ aggregation kinetics. (A) ThT aggregation assay showing the rapid oligomerization of 32 µM $A\beta_{1-40}$ in the presence of 24 µM thioflavin T (ThT) at pH 7.4 in DPBS, at 37 ºC, for 21 h. Amyloid formation was monitored by an increase in ThT fluorescence at 485 nm. Values are expressed as a percentage of the maximum ThT fluorescence. Graph represents the mean ± SEM (n = 3 independent experiments). **(B)** Electron micrograph demonstrating the rapid tendency of the peptide to aggregate and form amyloid fibrils after 21 hours in solution. Immunoreactivity was detected using an Au nanoparticle (10 nm) conjugated secondary antibody. Scale bar, 0.2 µm.

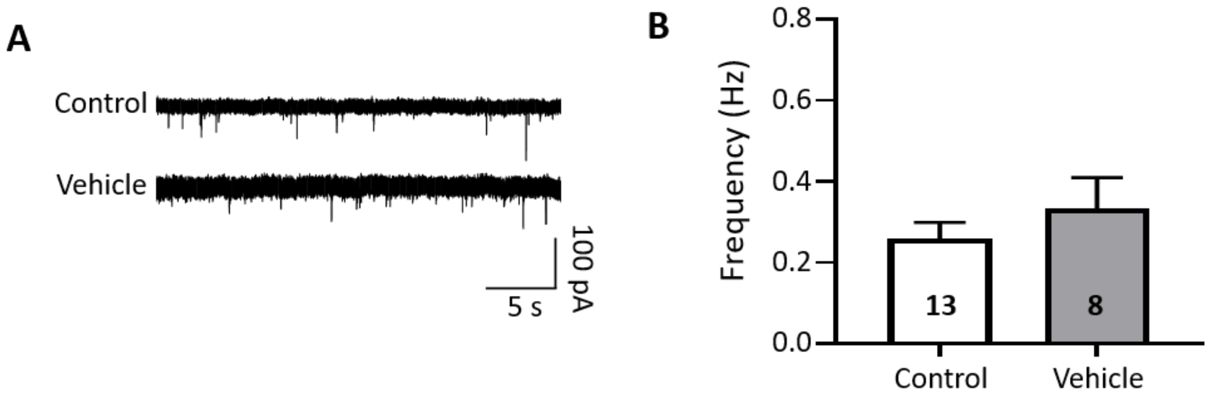

Supplementary Figure S2. Vehicle control for intracellular αSynO effects. (A, B) Representative miniature post-synaptic currents **(A)** and frequency quantification **(B)** for control and vehicle conditions. Control synaptic current recordings were made with an intracellular solution without αSynO, and the vehicle sample includes only the solvents used for the preparation of αSynO. Bars represent the mean ± SEM from the indicated number of neurons. Unpaired Student's t-test. Control 13 y vehiculo 8.

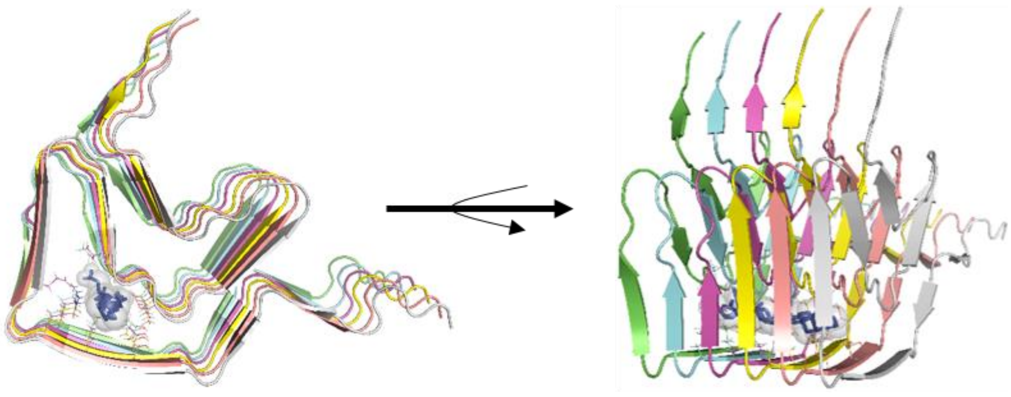

Supplementary Figure S3. Alternative position of M30 in the αSyn fibril predicted by protein-ligand docking. Each subunit is individually colored, and 4 predicted positions of M30 are shown inside the fiber with a binding site that varies mainly in the subunits that form it, conserving the amino acids involved.

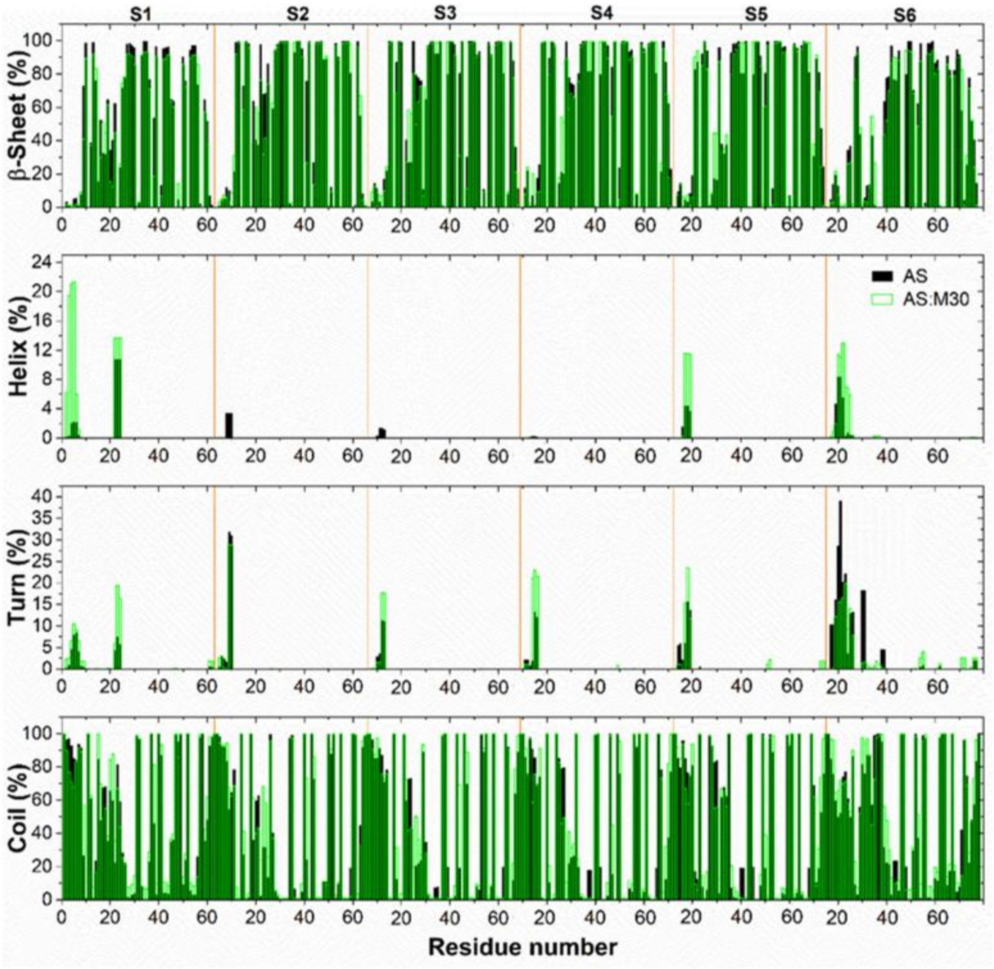

Supplementary Figure S4. Shifts in the secondary structure elements induced by M30 in αSyn fibrils. The average secondary structure per residue was calculated by averaging all αSyn and αSyn:M30 conformations acquired between 200 and 500 ns of each trajectory. Subunits are labelled on the top of the diagram.

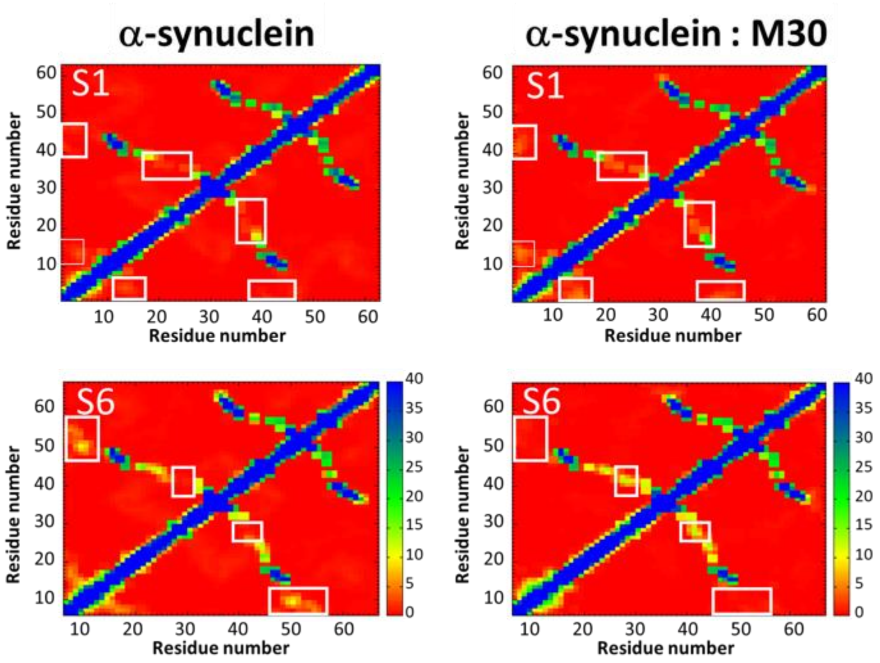

Supplementary Figure S5. Modifications of αSyn contact maps in the presence of M30. The intra-subunit residue contacts are displayed for αSyn and αSyn:M30 at subunit S1 and S6.

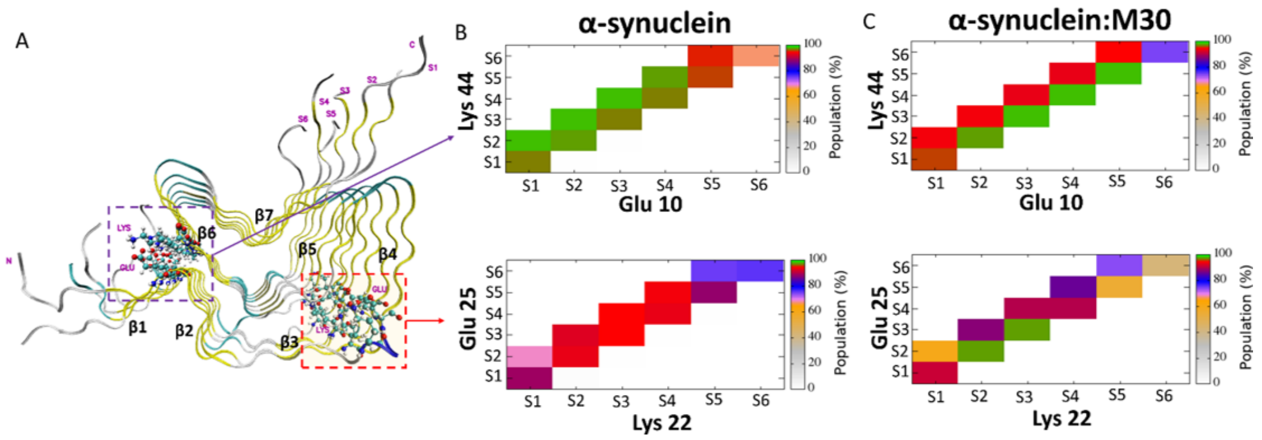

Supplementary Figure S6. Effects on salt bridge formation in αSyn by M30. (A) *In silico* simulations showing αSyn aggregates composed of six monomers. Each monomer possesses seven beta sheets forming short intra-peptide Lys22-Glu25 (segmented square in red) and long inter-peptide Glu10-Lys44 salt bridges (segmented square in purple). **(B)** Contact maps for αSyn showing intra-and inter-peptide salt bridges. **(C)** Variation of the same salt bridges in the presence of M30.
