# Supplementary material for: The Stimulatory Effects of Intracellular α-Synuclein on Synaptic Transmission Are Attenuated by 2-Octahydroisoquinolin-2(1H)-ylethanamine
Source: Int J Mol Sci. 2021 Dec 9;22(24):13253. doi: 10.3390/ijms222413253 (PMC8705949; doi:10.3390/ijms222413253)
Supplement: Supplementary file 1 [file ijms-22-13253-s001.zip › Supplementary Table S1.pdf]

| System | Subunit | β-sheet | α-helix | Turn | Coil |
|---|---|---|---|---|---|
| **αSyn** | S1 | 46.33 ± 0.50 | 0.63 ± 0.49 | 0.73 ± 0.33 | 52.29 ± 0.72 |
| | S2 | 54.89 ± 0.46 | 0.15 ± 0.13 | 1.11 ± 0.46 | 43.83 ± 0.75 |
| | S3 | 56.24 ± 0.76 | 0.06 ± 0.06 | 0.46 ± 0.32 | 43.22 ± 1.06 |
| | S4 | 56.28 ± 0.88 | 0.01 ± 0.01 | 0.52 ± 0.17 | 43.17 ± 0.81 |
| | S5 | 52.70 ± 0.72 | 0.22 ± 0.13 | 0.78 ± 0.29 | 46.28 ± 1.09 |
| | S6 | 42.78 ± 2.00 | 0.45 ± 0. 26 | 3.63 ± 0.61 | 53.12 ± 2.24 |
| | **S1-S6*** | **51.54 ± 0.68** | **0.25 ± 0.08** | **1.21 ± 0.14** | **46.98 ± 0.83** |
| **αSyn:M30** | S1 | 42.01 ± 1.73 | 1.85 ± 0.82 | 1.39 ± 0.62 | 54.74 ± 0.92 |
| | S2 | 52.52 ± 1.21 | 0.00 ± 0.00 | 1.01 ± 0.45 | 46.46 ± 1.63 |
| | S3 | 54.30 ± 1.19 | 0.00 ± 0.00 | 0.61 ± 0.27 | 45.07 ± 0.99 |
| | S4 | 54.72 ± 1.43 | 0.00 ± 0.00 | 1.10 ± 0.49 | 44.17 ± 2.04 |
| | S5 | 52.33 ± 1.07 | 0.55 ± 0.24 | 0.94 ± 0.42 | 46.16 ± 1.74 |
| | S6 | 40.46 ± 1.16 | 0.95 ± 0.42 | 2.19 ± 0.97 | 56.38 ± 1.78 |
| | **S1-S6*** | **49.39 ± 1.10** | **0.55 ± 0.08** | **1.21 ± 0.39** | **48.83 ± 1.25** |
| * Average secondary structure of αSyn and αSyn:M30 fibril (%) ± SEM | | | | | |

**Supplementary Table S1:** Average secondary structure content (%) ± SEM at each subunit for αSyn and αSyn:M30 fibril.
